# Dual SGLT1/2 inhibition with sotagliflozin improves cardiac function and metabolism in type 2 diabetic mice

**DOI:** 10.1038/s44324-026-00119-z

**Published:** 2026-08-05

**Authors:** Megan Young, Sanushi Dambure, Fenn Cullen, Kimia Jarrah, Lana McClements, Zenouska Ramchunder, Dunja Aksentijevic

**Affiliations:** 1 William Harvey Research Institute, Barts and the London Faculty of Medicine and Dentistry, Queen Mary University of London, London, UK; 2 School of Life Sciences & Institute for Biomedical Materials and Devices, Faculty of Science, University of Technology Sydney, Ultimo, Australia

**Keywords:** Cardiology, Diseases, Endocrinology, Physiology

## Abstract

Sotagliflozin (SOTA), a dual sodium-glucose cotransporter (SGLT)1/2 inhibitor, improves cardiovascular outcomes in patients with diabetes and heart failure, yet the metabolic mechanisms underlying these benefits remain incompletely defined. As a model of type 2 diabetes, db/db mice were treated with SOTA (5 mg/kg/day) in drinking water for 4 weeks. Cardiac metabolism was assessed using LC-MS/MS, cardiac function by echocardiography, and susceptibility to ischemia/reperfusion injury in Langendorff-perfused hearts. Systemic metabolism was characterised by plasma biochemical profiling and ^1^H NMR spectroscopy of peripheral tissues. db/db mice exhibited obesity, hyperglycaemia, hyperinsulinaemia, and diastolic dysfunction, alongside perturbed cardiac and systemic metabolism. SOTA treatment improved hyperglycaemia, ventricular function and cardiac metabolomic profile without correcting obesity or insulin resistance. In the heart, SOTA partially restored glycolytic intermediates and improved bioenergetics, whereas systemic metabolic abnormalities largely persisted. These findings suggest dual SGLT1/2 inhibition induces selective cardiac metabolic remodelling in diabetic cardiomyopathy independent of global metabolic correction.

## Introduction

Type 2 diabetes mellitus (T2DM) substantially increases the risk of heart failure and contributes to the development of diabetic cardiomyopathy, a condition characterised by structural remodelling and ventricular dysfunction in the absence of coronary artery disease or hypertension^1–3^. Diabetic cardiomyopathy is a myocardial disorder driven by metabolic disturbances including insulin resistance, altered substrate utilisation, mitochondrial dysfunction, oxidative stress, and myocardial fibrosis^4^. These processes lead to progressive ventricular remodelling, frequently manifesting as diastolic dysfunction and impaired ventricular compliance that can contribute to heart failure with preserved ejection fraction (HFpEF)^5,6^.

Sodium-glucose cotransporter (SGLT) inhibitors have emerged as a major therapeutic class of glucose-lowering agents with established cardiovascular benefits in patients with T2DM and heart failure^7,8^. Among these agents, sotagliflozin (SOTA) is unique as a dual inhibitor of SGLT2 in the kidney and SGLT1 in the intestine, thereby reducing renal glucose reabsorption and delaying intestinal glucose absorption^9^. In contrast to SGLT2, SGLT1 is expressed within the myocardium, making it particularly relevant to the pathophysiology of diabetic cardiomyopathy^10,11^. Therefore, compared with selective SGLT2 inhibitors, dual SGLT1/2 inhibition may offer additional metabolic and myocardial effects that could provide therapeutic advantages. Experimental evidence suggests that myocardial SGLT1 may contribute to the development and progression of diabetic cardiomyopathy^12^. Clinical trials have demonstrated significant reductions in hospitalisation for heart failure and cardiovascular events in patients treated with SGLT inhibitors, suggesting benefits that extend beyond glucose lowering^7,8^. The cardiovascular efficacy of SOTA was demonstrated in the SOLOIST-WHF trial, in which treatment significantly reduced the composite outcome of cardiovascular death and heart failure events in patients with T2DM and recent worsening heart failure^13^. Despite these clinical benefits, the mechanisms through which SOTA improves cardiovascular outcomes remain incompletely understood, including whether they are mediated through improvements in systemic glucose homeostasis or through alternative metabolic and cardiac remodelling pathways.

Beyond glucose lowering, SGLT inhibition has been proposed to exert pleiotropic cardioprotective effects through mechanisms including osmotic diuresis, modulation of inflammation and oxidative stress, and alterations in myocardial substrate utilisation. These drugs may improve myocardial energetics by shifting cardiac metabolism toward more efficient substrates such as ketone bodies and fatty acids. Such metabolic remodelling has been proposed as a key contributor to the cardiovascular benefits observed in both experimental models and clinical studies of SGLT inhibition^14–16^.

The db/db mouse, a leptin receptor-deficient model of T2DM, develops severe obesity, hyperglycaemia, insulin resistance, and cardiac structural and functional abnormalities resembling diabetic cardiomyopathy^1,5^. This model therefore provides an experimental platform for studying metabolic and cardiac effects of antidiabetic therapies^17^.

**Fig. 1:**
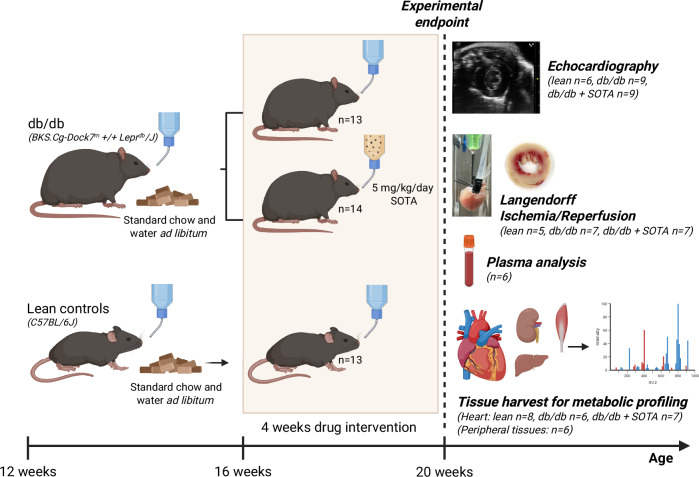
Schematic summary of the study protocol. Male db/db mice (*n* = 27 total: untreated db/db *n* = 13, SOTA treated db/db *n* = 14). C57BL/6 J lean control mice (*n* = 13). Schematic created in Biorender.com.

In the present study, using echocardiography, plasma biochemistry, and targeted metabolomic profiling (Fig. 1), we investigated the effects of 4-weeks of SOTA treatment on cardiac structure, ventricular function, renal safety, and systemic metabolism in db/db mice. We aimed to determine whether the cardioprotective effects of SOTA are associated with improvements in systemic metabolic status or reflect alterations in cardiac metabolic and structural remodelling.

## Results

### Morphological and metabolic characteristics of the experimental model

**Fig. 2:**
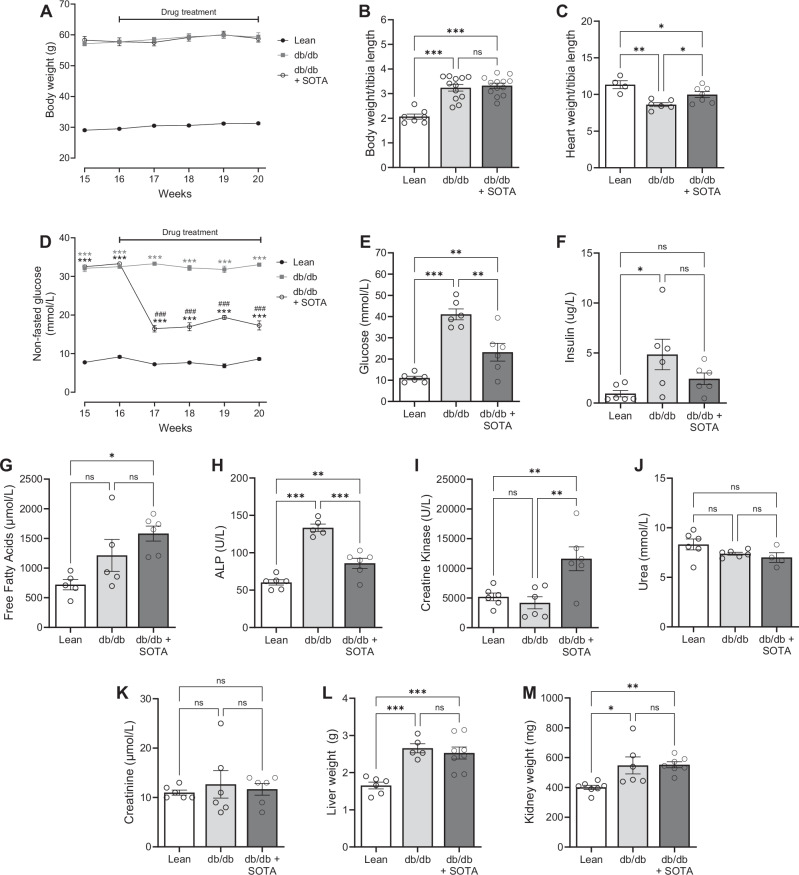
Morphological characteristics and plasma metabolite profile of db/db mice post- SOTA treatment. **A** Body weight monitoring. Lean (*n* = 10), db/db (*n* = 12), db/db + SOTA (*n* = 13). **B** Terminal body weight normalised to tibia length. Analysed by one-way ANOVA with Holm-Šídák post-hoc analysis displayed, *p < 0.05. Lean (*n* = 7), db/db (*n* = 12), db/db + SOTA (*n* = 13). **C** Heart weight normalised to tibia length. Analysed by one-way ANOVA with Holm-Šídák post-hoc analysis displayed, *p < 0.05. Lean (*n* = 4), db/db (*n* = 6), db/db + SOTA (*n* = 7). **D** Non-fasted glucose measurements. Analysed by two-way repeated measures ANOVA, significant interaction of time x group (*p* < 0.001), post-hoc comparisons using Fisher’s LSD test displayed for each time point (*p < 0.05 vs. lean; #*p* < 0.05 vs. db/db + SOTA). Lean (*n* = 5), db/db (*n* = 6), db/db + SOTA (*n* = 7). **E–K** Plasma concentrations of (**E**) glucose, (**F**) Insulin, (**G**) Free fatty acids, (**H**) Alkaline phosphatase (ALP), (**I**) Creatine kinase, (**J**) Urea and (**K**) Creatinine measured in terminal blood samples at 20-weeks of age. Analysed by one-way ANOVA with Holm-Šídák post-hoc analysis displayed (**E**, **G**–**J**) or Kruskal-Wallis test with Dunn’s post-hoc analysis displayed (**F**, **K**), *p < 0.05. Lean, *n* = 6 (FFAs, *n* = 5); db/db *n* = 6 (FFAs and ALP, *n* = 5); db/db + SOTA, *n* = 6 (Urea, *n* = 4). **L** Liver weight. Analysed by one-way ANOVA with Holm-Šídák post-hoc analysis displayed, *p < 0.05. Lean (*n* = 6), db/db (*n* = 5), db/db + SOTA (*n* = 8). **M** Kidney weight. Analysed by Kruskal-Wallis test with Dunn’s post-hoc analysis displayed, *p < 0.05. Lean (*n* = 7), db/db (n = 6), db/db + SOTA (*n* = 7).

Baseline morphological and metabolic characteristics of the experimental groups are summarised in Fig. 2. As expected, db/db mice exhibited a pronounced obese phenotype compared with lean controls (Fig. 2A), terminal body weight normalised to tibia length was significantly increased in db/db animals (Fig. 2B). Despite their increased body mass, db/db mice displayed smaller hearts relative to lean controls (Fig. 2C), consistent with previous observations from cine MRI-based geometric assessments of cardiac structure in this model^1^.

In addition to obesity, db/db mice exhibited severe metabolic disturbances. Circulating glucose levels were markedly elevated compared with lean animals, confirming the presence of severe hyperglycaemia (Fig. 2D, E). Plasma insulin concentrations were also substantially increased (Fig. 2F), consistent with systemic insulin resistance. Circulating lipid levels in db/db mice were increased by 68% compared to lean controls (Fig. 2G). Markers of hepatic dysfunction were also altered in db/db mice. Plasma alkaline phosphatase (ALP) levels were significantly increased compared with lean controls, suggesting liver injury or metabolic liver dysfunction (Fig. 2H). Circulating creatine kinase (CK), a marker of muscle injury, was not significantly different between lean and db/db mice (Fig. 2I). Renal function of db/db mice was comparable to controls (Fig. 2J, K). Consistent with systemic metabolic alterations, db/db animals also exhibited increased liver and kidney weights relative to lean controls (Fig. 2L, M).

Four weeks of SOTA treatment did not significantly alter body weight in db/db mice, indicating that treatment did not reverse the obese phenotype (Fig. 2A, B). Although SOTA treatment partially increased HW/TL in db/db mice, treated hearts remained significantly smaller than those of lean animals (Fig. 2C). Liver and kidney weights remained elevated and were not significantly changed by SOTA treatment (Fig. 2L, M). Drug treatment resulted in a marked improvement in glycaemic control (Fig. 2D). Plasma glucose levels were dramatically reduced at the end of 4-weeks SOTA treatment although remained elevated compared to lean controls (Fig. 2E). Circulating insulin levels were not significantly improved by SOTA treatment, although a modest reduction was observed compared to untreated db/db mice, however, levels remained higher than controls, consistent with persistent hyperinsulinaemia (Fig. 2F). Plasma lipid concentrations also remained elevated following SOTA administration, suggesting that hyperlipidaemia was not corrected by the treatment (Fig. 2G). With respect to liver function markers, SOTA administration significantly reduced plasma ALP levels relative to untreated db/db mice, indicating partial improvement in markers of hepatic injury (Fig. 2H). Circulating CK levels were increased in SOTA-treated animals compared with both lean controls and untreated db/db mice (Fig. 21). Urea and creatinine remained unaffected by SOTA drug treatment (Fig. 2J, K).

### Echocardiographic assessment of cardiac structure and function

**Fig. 3:**
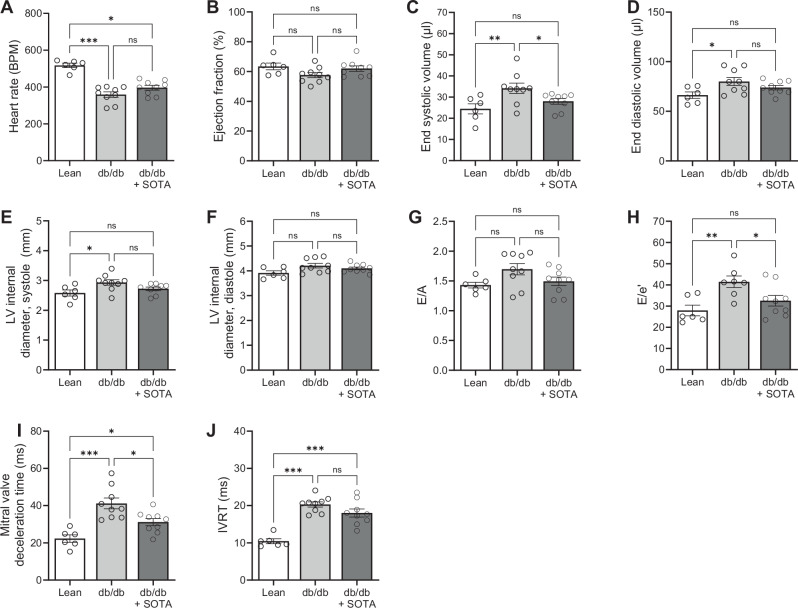
Cardiac function assessed by echocardiography. **A** Heart rate, (**B**) Ejection fraction, (**C**) end systolic volume, (**D**) end diastolic volume, (**E-F**) left ventricular (LV) internal diameter in systole (**E**) and diastole (**F**), (**G**) E/A ratio, (**H**) E/e’ ratio, (**I**) mitral valve deceleration time and (**J**) isovolumetric relaxation time (IVRT). Analysed by one-way ANOVA with Holm-Šídák post-hoc analysis displayed (**B**–**J**) or Kruskal-Wallis test with Dunn’s post-hoc analysis displayed (**A**), *p < 0.05. Lean *n* = 6, db/db n = 9 (E/e’ *n* = 7), db/db + SOTA *n* = 9.

Transthoracic echocardiography was performed to evaluate cardiac structure and function in lean controls, untreated db/db mice, and SOTA-treated db/db mice (Fig. 3). Heart rate was lower in db/db mice compared with lean controls (Fig. 3A). Untreated db/db mice demonstrated preserved left ventricular (LV) systolic function, with ejection fraction not significantly different from lean controls (Fig. 3B). However, structural and volumetric differences were observed. db/db hearts exhibited significantly increased end systolic and diastolic volumes alongside increased LV internal diameter (Fig. 3C–F), consistent with LV dilation. However, LV wall thicknesses were unchanged (Fig. S1). Assessment of diastolic function revealed significant alterations in ventricular filling parameters in db/db mice. Transmitral E/A ratio was slightly elevated in untreated db/db mice (Fig. 3G), while the E/e′ ratio was significantly increased compared with lean controls (Fig. 3H) indicative of impaired diastolic function. Furthermore, mitral valve (MV) deceleration time (Fig. 3I) and isovolumetric relaxation time (IVRT, Fig. 3J) were significantly prolonged, consistent with diastolic dysfunction.

Following 4 weeks of SOTA treatment, several echocardiographic parameters were improved in db/db mice. End-systolic volume was significantly reduced to levels comparable with lean controls (Fig. 3C), and LV internal diameter in SOTA-treated db/db mice trended towards the levels observed in lean controls (Fig. 3E). SOTA treatment alleviated the changes in both the E/e’ ratio and MV deceleration time seen in untreated db/db mice, indicating improvement in diastolic function (Fig. 3H, I).

### Cardiac metabolomic profile of db/db hearts

**Fig. 4:**
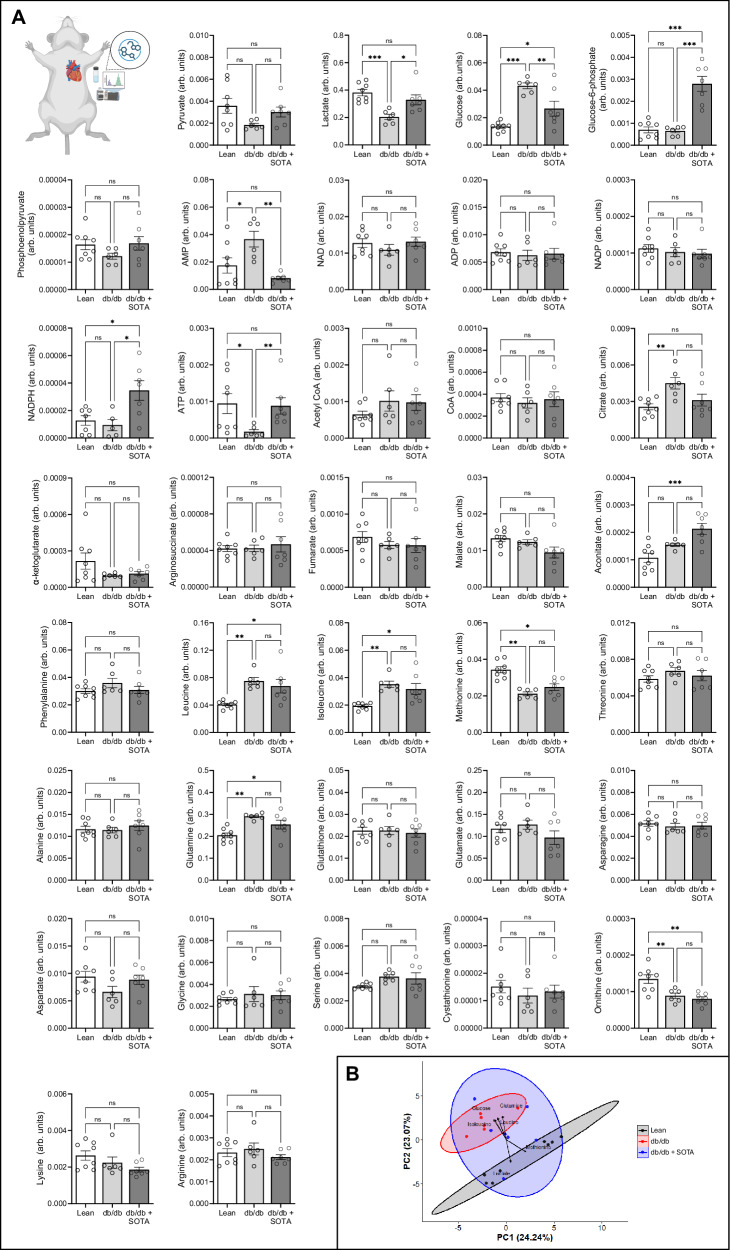
Cardiac metabolomic profile of db/db mice following SOTA treatment assessed by liquid chromatography-mass spectrometry (LC-MS). **A** Analysed by one-way ANOVA with Holm-Šídák post-hoc analysis displayed or Kruskal-Wallis test with Dunn’s post-hoc analysis displayed (ADP, ATP, acetyl CoA, leucine, isoleucine, methionine, glycine, cystathionine), *p < 0.05. Data are shown as individual points with mean ± SEM. Lean, *n* = 8 (NADPH, *n* = 7); db/db *n* = 6 (NADPH, *n* = 5); db/db + SOTA, *n* = 7. Schematic created in Biorender.com. **B** Biplot illustrating metabolites contributing to metabolomic profile. Each point represents an individual biological sample, and clustering reflects similarities in metabolomic profiles. The direction and length of vectors indicate the contribution and influence of individual metabolites to group separation.

Targeted LC-MS/MS metabolomic analysis revealed that the several metabolites involved in glycolysis, the tricarboxylic acid (TCA) cycle, and amino acid metabolism were significantly altered in db/db hearts (Fig. 4A). Within the glycolytic pathway, myocardial glucose levels were markedly elevated in db/db hearts (Fig. 4A). In contrast, downstream glycolytic intermediates were reduced, with significantly lower levels of lactate detected compared to lean controls, whilst pyruvate and phosphoenolpyruvate levels showed a trend towards reduction (Fig. 4A). Together, these findings indicate substantial perturbation of glycolysis in the diabetic myocardium. Alterations were also observed in TCA cycle and bioenergetic intermediates. AMP levels were markedly increased in db/db hearts, and ATP levels were significantly decreased (Fig. 4A), consistent with altered myocardial energetic status. Citrate levels were substantially elevated, while aconitate levels were markedly increased (*p* = 0.057) (Fig. 4A). Total CoA levels were not significantly different between groups (Fig. 4A). Amino acid profiling revealed pronounced changes in branched-chain amino acids and several other amino acid metabolites (Fig. 4A). Leucine and isoleucine, as well as glutamine and serine, levels were significantly elevated in db/db hearts. In contrast, methionine and ornithine were reduced relative to control levels (Fig. 4A). Overall, these metabolomic alterations demonstrate substantial remodelling of myocardial metabolic pathways in untreated db/db mice (Fig. 4B, PCA plot summary), affecting glycolytic intermediates, TCA cycle metabolites, and multiple amino acid pools.

Within the glycolytic pathway, several metabolites that were altered in untreated db/db hearts exhibited levels comparable to lean controls following SOTA treatment (Fig. 4A). Myocardial lactate levels, which were reduced in untreated db/db mice, returned to levels comparable to lean controls (Fig. 4A). Myocardial glucose concentrations were reduced by 39.5% compared with untreated db/db hearts, although glucose levels remained elevated compared with lean animals. Glucose-6-phosphate levels were markedly increased following SOTA treatment (Fig. 4A). Substantial changes were also observed in myocardial energetic metabolites (Fig. 4A). AMP levels, which were markedly elevated in untreated db/db hearts, were reduced following SOTA administration. ATP levels, which were reduced in untreated diabetic hearts, were restored and comparable to lean controls following SOTA treatment. In addition, NADPH concentrations were markedly increased, exhibiting a 3.7-fold increase relative to untreated db/db hearts (Fig. 4A). Alterations in TCA cycle intermediates were also detected following treatment. Citrate levels, which were elevated in untreated db/db mice, were reduced and comparable with lean controls post-SOTA treatment (Fig. 4A). Aconitate levels were increased to a greater extent relative to lean controls than in untreated db/db hearts (Fig. 4A). Total CoA levels remained unchanged and were comparable to those observed in untreated animals, and other measured TCA cycle intermediates did not show substantial changes with treatment. In contrast to the changes observed in carbohydrate and TCA cycle metabolites, SOTA treatment had limited effects on myocardial amino acid profiles (Fig. 4A). The elevations in branched-chain amino acids and other amino acid alterations observed in untreated db/db hearts were largely unchanged following treatment. Among the amino acids changed in untreated db/db hearts, only serine and glutamine levels exhibited no difference to lean controls following SOTA treatment (Fig. 4A).

### Cardiac functional recovery and infarct size following ischemia/reperfusion

To assess whether SOTA treatment alters susceptibility to myocardial ischemia/reperfusion injury, isolated hearts from control, untreated db/db, and SOTA-treated db/db mice were subjected to global ischemia/reperfusion using the Langendorff perfusion model. Hearts were exposed to 20 minutes of total global ischemia followed by reperfusion, and post-ischemic functional recovery was assessed.

**Fig. 5:**
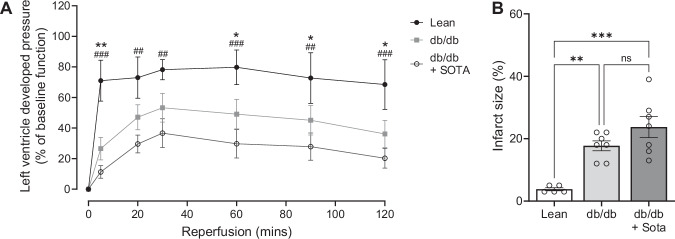
Susceptibility to myocardial ischemia reperfusion injury. **A** Recovery of left ventricle developed pressure during reperfusion following 20 min global ischemia. Analysed by two-way repeated measures ANOVA, significant interaction of time x group (*p* = 0.05), post-hoc comparisons using Fisher’s LSD test displayed for each time point (*p < 0.05 lean vs. db/db; #*p* < 0.05 lean vs. db/db + SOTA). Lean (*n* = 4), db/db (*n* = 7), db/db + SOTA (*n* = 7). **B** Quantification of infarct size at the end of reperfusion, infarct size expressed as a percentage of total tissue area. Analysed by one-way ANOVA with Holm-Šídák post-hoc analysis displayed, *p < 0.05. Lean (*n* = 5), db/db (*n* = 7), db/db + SOTA (*n* = 7).

Untreated db/db hearts demonstrated impaired functional recovery following ischemia compared with control hearts. Measures of post-ischemic cardiac performance (left ventricular developed pressure, dP/dt max) during reperfusion were markedly reduced in db/db hearts (Fig. 5A, Fig. S2), indicating greater susceptibility to ischemia/reperfusion injury in the diabetic myocardium. Consistent with the functional data, infarct size assessed by TTC staining was significantly greater in untreated db/db hearts compared with control hearts (Fig. 5B), demonstrating increased myocardial injury following ischemia/reperfusion in diabetic animals.

Despite the metabolic changes observed following four weeks of SOTA treatment, no significant improvement in ischemia/reperfusion outcomes was detected in treated db/db hearts. Post-ischemic functional recovery was not improved compared to that observed in untreated db/db hearts (Fig. 5A, Fig. S2), and infarct size measured by TTC staining was not significantly different between treated and untreated diabetic groups (Fig. 5B).

### Liver, kidney and muscle metabolome

**Fig. 6:**
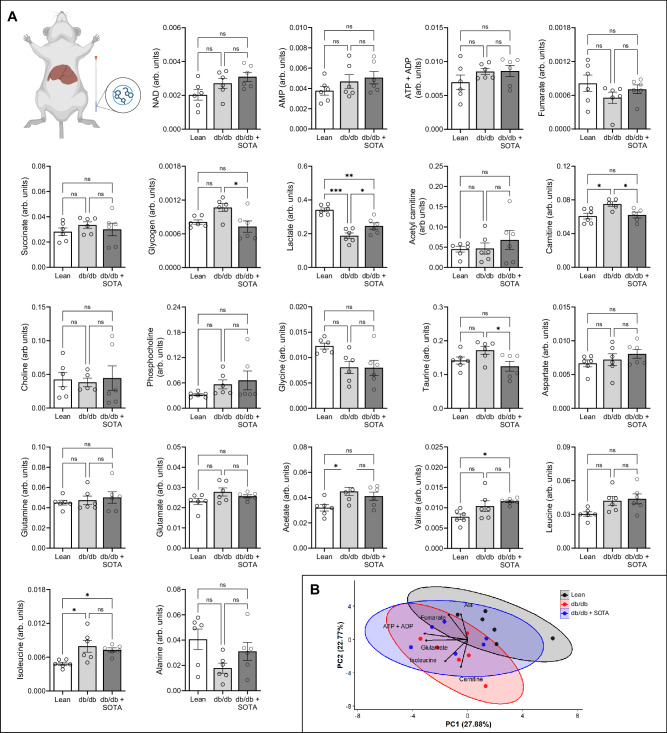
Liver metabolomic profile of db/db mice following SOTA treatment measured by ^1^H NMR spectroscopy. **A** Analysed by one-way ANOVA with Holm-Šídák post-hoc analysis displayed or Kruskal-Wallis test with Dunn’s post-hoc analysis displayed (AMP, phosphocholine), *p < 0.05. Data are shown as individual points with mean ± SEM. Lean, *n* = 6; db/db *n* = 6 (carnitine and choline, *n* = 5); db/db + SOTA, *n* = 6 (carnitine, *n* = 5). Schematic created in Biorender.com. **B** Biplot illustrating metabolites contributing to metabolomic profile. Each point represents an individual biological sample, and clustering reflects similarities in metabolomic profiles. The direction and length of vectors indicate the contribution and influence of individual metabolites to group separation.

**Fig. 7:**
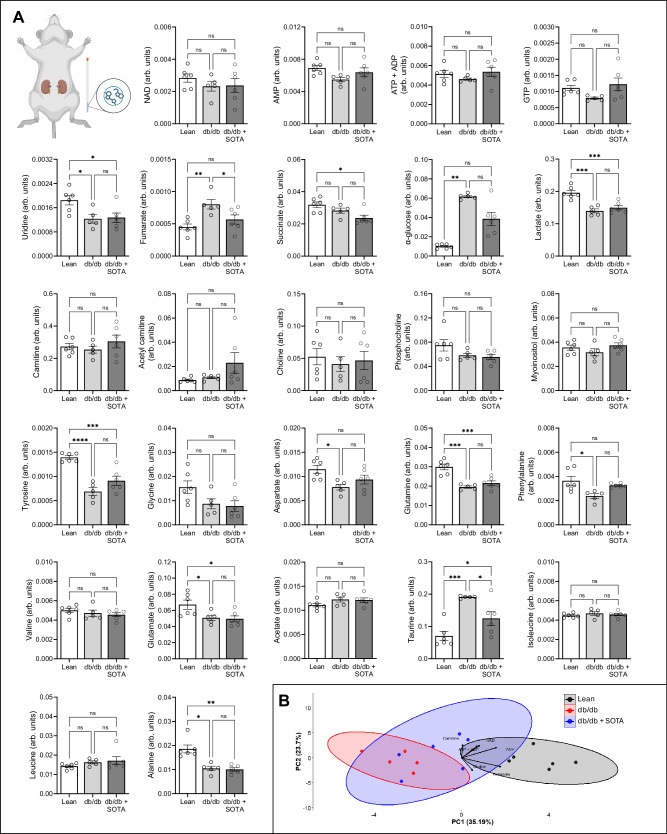
Kidney metabolomic profile of db/db mice following SOTA treatment measured by ^1^H NMR spectroscopy. **A** Analysed by one-way ANOVA with Holm-Šídák post-hoc analysis displayed or Kruskal-Wallis test with Dunn’s post-hoc analysis displayed (α-glucose, acetyl carnitine, choline, alanine, leucine), *p < 0.05. Data are shown as individual points with mean ± SEM. Lean, *n* = 6; db/db *n* = 5; db/db + SOTA, *n* = 6. Schematic created in Biorender.com. **B** Biplot illustrating metabolites contributing to metabolomic profile. Each point represents an individual biological sample, and clustering reflects similarities in metabolomic profiles. The direction and length of vectors indicate the contribution and influence of individual metabolites to group separation.

**Fig. 8:**
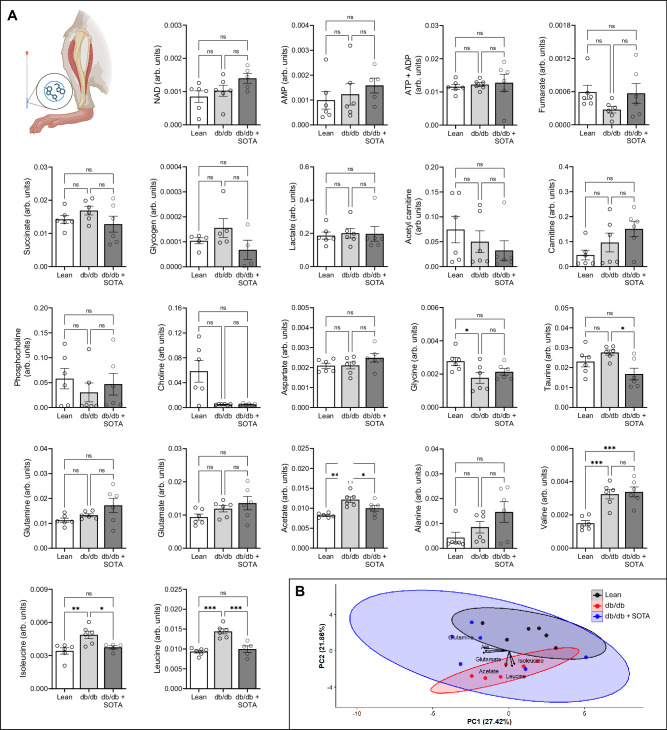
Muscle metabolomic profile of db/db mice following SOTA treatment measured by ^1^H NMR spectroscopy. Analysed by one-way ANOVA with Holm-Šídák post-hoc analysis displayed or Kruskal-Wallis test with Dunn’s post-hoc analysis displayed (), *p < 0.05. Data are shown as individual points with mean ± SEM. Lean, *n* = 6 (glycogen, *n* = 5); db/db *n* = 6 (glycogen, *n* = 5); db/db + SOTA, *n* = 6 (NAD, AMP, leucine, isoleucine, *n* = 5; glycogen, *n* = 4). Schematic created in Biorender.com. **B** Biplot illustrating metabolites contributing to metabolomic profile. Each point represents an individual biological sample, and clustering reflects similarities in metabolomic profiles. The direction and length of vectors indicate the contribution and influence of individual metabolites to group separation.

Comparison of metabolite profiles between db/db and lean animals revealed tissue-specific metabolic perturbations affecting multiple metabolic pathways (Figs. 6–8). Analysis of liver tissue extracts revealed several significant differences in metabolite abundance between db/db and lean animals including elevated levels of acetate, carnitine and isoleucine (Fig. 6A). Glycogen levels were also elevated in db/db animals (*p* = 0.05), indicating altered hepatic carbohydrate storage. In contrast, lactate levels were reduced in db/db liver tissue (Fig. 6A). Metabolomic analysis of kidney tissue demonstrated multiple alterations in db/db mice compared with lean animals (Fig. 7A). Fumarate, α-glucose and taurine levels were increased in diabetic kidneys, whilst several metabolites (uridine, lactate, tyrosine, phenylalanine, glutamine, aspartate, glutamate and alanine) were reduced relative to lean controls (Fig. 7A). In skeletal muscle tissue, db/db mice exhibited increased levels of branched-chain amino acids leucine, valine, and isoleucine (Fig. 8A). Acetate levels were also increased in diabetic skeletal muscle whilst glycine levels were decreased compared to lean controls (Fig. 8A). Together, these findings demonstrate widespread metabolic remodeling across multiple peripheral tissues in db/db mice, with tissue-specific alterations observed in carbohydrate metabolism and amino acid metabolism.

Comparison of SOTA-treated db/db animals with lean controls revealed that several peripheral tissues metabolite abnormalities persisted following treatment, although the number of significantly altered metabolites was reduced in all tissues examined. In treated db/db livers, branched-chain amino acid isoleucine remained elevated whilst glycine levels remained reduced (Fig. 6A). SOTA treatment normalised the elevated levels of carnitine and partially ameliorated the reduction in lactate observed in untreated db/db livers. Furthermore, glycogen and taurine levels were reduced compared to the slight elevation observed in untreated db/db liver (Fig. 6A). Kidney metabolomic analysis revealed persistent metabolic alterations despite SOTA treatment, several metabolites remained significantly different from lean controls (uridine, succinate, lactate, tyrosine, glutamine, glutamate, alanine) (Fig. 7A). The observed changes in fumarate, phenylalanine, aspartate and taurine in untreated db/db kidneys were improved following SOTA treatment (Fig. 7A). In skeletal muscle tissue, SOTA treatment ameliorated the accumulation of acetate, isoleucine and leucine observed in untreated db/db mice (Fig. 8A).

To assess overall metabolic variations between experimental groups across tissues, principal component analysis (PCA) was performed as an unsupervised multivariate approach (Figs. 6–8). PCA demonstrated clear separation between lean and db/db animals in kidney and skeletal muscle tissues, indicating substantial metabolic remodelling associated with diabetes (Figs. 7, 8). In all tissues, SOTA-treated db/db animals exhibited a partial shift toward the lean metabolic profile, consistent with the partial normalisation of several metabolites observed in the univariate analyses. In liver, PCA revealed broader overlap between groups (Fig. 6), although db/db animals remained distinguishable from lean controls, and SOTA treatment partially shifted the metabolic phenotype. Together, these multivariate analyses support the presence of pronounced tissue-specific metabolic disturbances in db/db mice and indicate that SOTA treatment improves, but does not fully normalise, systemic metabolic remodelling across peripheral organs.

## Discussion

The present study demonstrates that 4-weeks of SOTA treatment improves cardiac structural remodelling and myocardial metabolic balance in type 2 diabetic mice despite persistent systemic insulin resistance. Using integrated echocardiographic, metabolomic, and functional analyses, we show that SOTA selectively restores key metabolic pathways in the diabetic heart while leaving broader systemic metabolic abnormalities largely unchanged.

The systemic phenotype observed in db/db mice in the present study is consistent with the well-established metabolic profile of this model of T2DM, characterised by severe obesity, hyperglycaemia, hyperinsulinaemia, dyslipidaemia, and evidence of hepatic dysfunction^1,5^. These abnormalities arise from leptin receptor deficiency and profound insulin resistance, resulting in widespread metabolic stress affecting multiple organs including liver, kidney, and heart. In agreement with previous reports, db/db animals displayed marked hyperglycaemia together with elevated circulating insulin and lipid levels, increased liver and kidney weights, and elevated circulating markers of hepatic injury^1,5^.

Four weeks of SOTA treatment produced robust improvement of circulating glucose levels but had limited effects on other systemic metabolic abnormalities. Body weight, liver weight, and kidney weight remained elevated, and both hyperinsulinaemia and hyperlipidaemia persisted following treatment. Together, these findings indicate substantial glycaemic improvements with limited correction of broader systemic metabolic abnormalities. Therefore, the observed metabolic and cardiac effects induced by SOTA in this model are partially driven by glycaemic control rather than correction of systemic insulin resistance or lipid metabolism. Similar dissociation between glucose-lowering and persistent hyperinsulinaemia has been reported with SGLT inhibition in both experimental models and clinical studies, where increased urinary glucose excretion reduces circulating glucose independently of insulin signalling^18^.

Circulating free fatty acid levels remained elevated and were not reduced following SOTA treatment. This may reflect increased lipolysis secondary to glycosuria-induced energy loss, particularly in the setting of persistent insulin resistance, where suppression of adipose tissue lipolysis is impaired. As a result, increased fatty acid mobilisation may contribute to elevated circulating lipid levels despite improved glycaemic control^19^. These findings further highlight the dissociation between glucose-lowering and broader metabolic abnormalities in this model. The differential effects of SOTA on glucose and lipid metabolism may also be influenced by disease stage. In db/db mice, dyslipidaemia develops early and progressively worsens with age. Although all groups in the present study were age-matched and lipid abnormalities were already established at the time of treatment, age-related disease progression may influence treatment responsiveness. Nevertheless, the lack of improvement in hyperlipidaemia despite improved glycaemic control suggests that lipid metabolism may require longer treatment duration or more extensive metabolic reprogramming than glucose homeostasis.

Interestingly, SOTA treatment was associated with a reduction in ALP, suggesting improvement in markers of hepatic injury^20^. The increase in circulating creatine kinase observed following treatment was unexpected and may reflect altered muscle metabolism or increased substrate mobilisation associated with glycosuric therapy, although the physiological significance of this observation remains uncertain.

Taken together, these findings indicate that short-term SOTA treatment significantly improves hyperglycaemia in db/db mice but does not substantially reverse the broader metabolic disturbances associated with severe insulin resistance. The persistence of hyperinsulinaemia, dyslipidaemia, and organ hypertrophy highlights the multifactorial nature of metabolic dysfunction in T2DM.

Diabetic cardiomyopathy is increasingly recognised as a distinct myocardial disorder characterised by structural and functional abnormalities that develop independently of coronary artery disease or hypertension. The natural history of diabetic cardiomyopathy typically involves early subclinical myocardial remodelling marked by increased fibrosis, impaired ventricular compliance, and diastolic dysfunction, with many patients developing HFpEF. However, HFpEF and systolic dysfunction leading to HFrEF are now recognised as distinct entities that do not necessarily represent sequential stages of disease progression^21^. Clinically, LV diastolic dysfunction represents the most common echocardiographic abnormality detected in asymptomatic individuals with diabetes who maintain a preserved ejection fraction^22^. More recent conceptual frameworks have also emphasised that diabetic cardiomyopathy reflects a disorder of myocardial metabolism, in which disturbances in substrate utilisation and mitochondrial energetics contribute directly to impaired myocardial relaxation and ventricular compliance^22^.

Consistent with these concepts, echocardiographic assessment in the present study demonstrated that db/db mice have preserved systolic function while exhibiting significant alterations in LV geometry consistent with LV dilatation, together with clear evidence of diastolic dysfunction. Findings align with the early HFpEF-like phenotype commonly observed in diabetic cardiomyopathy, with functional impairment manifesting as abnormalities in ventricular filling and relaxation rather than overt systolic failure. Diabetes-associated metabolic stress, insulin resistance, and lipid accumulation are known to promote myocardial remodelling through mechanisms involving mitochondrial dysfunction, impaired substrate flexibility, and cardiomyocyte structural alterations^3,4,22^. Importantly, SOTA treatment ameliorated structural remodelling and functional abnormalities in diabetic hearts. Improvements in LV geometry were reflected by reduced end-systolic volume, and indices of diastolic function were significantly improved, indicating attenuation of diabetic cardiac remodelling. Growing evidence indicates that SGLT inhibition improves cardiac structure and function through multiple mechanisms, including reductions in glucotoxicity, improvements in myocardial energetics, and alterations in substrate utilisation. The preservation of ejection fraction in both untreated and treated animals suggests that the observed improvements are more consistent with modulation of early remodelling processes occurring prior to overt systolic dysfunction.

The metabolomic profile observed in untreated db/db hearts is consistent with the metabolic remodelling that characterises diabetic cardiomyopathy. Markedly elevated myocardial glucose levels accompanied by reduced downstream glycolytic intermediates, including lactate, pyruvate, and phosphoenolpyruvate, suggest impaired glycolytic flux despite intracellular glucose accumulation. This pattern reflects the metabolic inflexibility characteristic of the diabetic heart, where impaired glucose oxidation and increased reliance on fatty acid metabolism disrupt the coupling between glycolysis and mitochondrial oxidative metabolism^4,22–24^.

Alterations in TCA cycle intermediates further support the presence of mitochondrial metabolic perturbations. The observed elevation in citrate and aconitate, together with reduced α-ketoglutarate suggests disruption of TCA cycle flux and altered mitochondrial substrate utilisation. Such mitochondrial dysfunction is widely recognised as a central feature of diabetic cardiomyopathy and contributes to impaired ATP generation and energetic stress in the diabetic myocardium^4,22–24^. While citrate accumulation may reflect altered coupling between glycolysis and mitochondrial metabolism, an additional explanation may involve increased fatty acid-derived acetyl-CoA input that is not fully matched by downstream TCA cycle processing. Such a mechanism would be consistent with the established metabolic inflexibility of diabetic cardiomyopathy, where reduced glucose oxidation is accompanied by increased reliance on fatty acid utilisation^5,6,22^. Previous in vivo hyperpolarized ^13^C NMR studies in 20-week db/db model have also demonstrated markedly reduced pyruvate dehydrogenase flux together with broader alterations in cardiac metabolic flux^1^, supporting a state of altered substrate utilisation and metabolic remodelling. While SGLT inhibition has been associated with transcriptional upregulation of myocardial fatty acid metabolism pathways^25^ and broader shifts in systemic substrate utilisation^26^, direct evidence that SOTA enhances myocardial fatty acid β-oxidation remains limited.

Amino acid metabolism was also markedly altered in db/db hearts, with accumulation of branched-chain amino acids, including leucine and isoleucine. Impaired branched-chain amino acid catabolism has emerged as a hallmark of metabolic disease and insulin resistance, reflecting dysregulated mitochondrial metabolism and altered substrate utilisation^27^. Because branched-chain amino acids provide carbon substrates for the TCA cycle, disruption of their catabolism may further contribute to metabolic inflexibility and mitochondrial dysfunction in the diabetic heart^28^.

Ketone body metabolism has also been proposed as a contributor to the cardioprotective effects of SGLT2 inhibition, providing an energetically efficient substrate for the myocardium^16,29,30^. Whether dual SGLT1/2 inhibition with sotagliflozin similarly alters ketone availability or myocardial ketone utilisation was not examined in the present study. Given the distinct pharmacological profile of SOTA, including intestinal SGLT1 inhibition and potential effects on myocardial glucose handling, its impact on ketone metabolism may differ from that of selective SGLT2 inhibitors. This represents an important area for future investigation.

One of the most prominent metabolic effects of SOTA treatment was the normalisation of glycolytic intermediates that were reduced in untreated db/db hearts. Lactate, pyruvate, and phosphoenolpyruvate levels were restored, while myocardial glucose accumulation was reduced. These changes suggest partial restoration of glycolytic processing and improved coupling between glycolysis and mitochondrial metabolism.

SOTA treatment also improved myocardial energetic metabolites. Reduction of AMP together with restoration of ATP levels suggests improvement in myocardial energetic status relative to untreated diabetic hearts. Energetic impairment is a defining feature of diabetic cardiomyopathy and has been closely linked to mitochondrial dysfunction and excessive reliance on fatty acid oxidation^22,24^.

Although SGLT1 is expressed in cardiomyocytes, myocardial glucose uptake under physiological conditions is primarily mediated by GLUT transporters. However, increasing evidence suggests that myocardial SGLT1 may become more relevant under pathological conditions, including diabetes and heart failure^12,31^. Marked changes in glucose-related metabolites observed following SOTA treatment are unlikely to reflect altered glucose transport alone and may instead indicate broader restoration of metabolic flux and improved coupling between glycolysis and mitochondrial metabolism. Improved engagement of glucose-dependent pathways may contribute to the partial restoration of metabolic flexibility observed in the diabetic myocardium.

A notable finding was the substantial increase in myocardial NADPH following SOTA treatment. NADPH plays a central role in maintaining cellular redox balance through antioxidant systems such as glutathione and thioredoxin. Increased NADPH availability may therefore enhance the capacity of the myocardium to counteract oxidative stress, which is a major contributor to diabetic cardiomyopathy^32^. Despite these improvements in carbohydrate metabolism and energetic status, SOTA had minimal effects on the altered amino acid profile of db/db hearts. Elevations in branched-chain amino acids persisted following treatment, suggesting that abnormalities in amino acid metabolism are less responsive to short-term improvements in glycaemic control. This persistence indicates that while SOTA can improve selected metabolic pathways, broader metabolic remodelling in diabetic cardiomyopathy may require longer treatment duration or additional interventions.

Consistent with previous studies of diabetic cardiomyopathy, untreated db/db hearts demonstrated markedly impaired functional recovery following global ischemia together with increased infarct size compared with control hearts^1,5^. Diabetes is known to increase susceptibility to ischemia/reperfusion injury through mechanisms including mitochondrial dysfunction, impaired metabolic flexibility, oxidative stress, and disrupted calcium handling^5^. Despite the metabolic improvements observed following SOTA treatment, and despite protective effects reported in pigs^33^, in our study, no improvement in post-ischemic functional recovery or infarct size was detected in treated db/db hearts.

Importantly, no significant differences were observed between untreated and SOTA-treated db/db hearts, indicating that SOTA neither improved nor worsened susceptibility to ischaemia/reperfusion (I/R) injury in this model. Although SGLT inhibition has frequently been associated with cardioprotection in experimental I/R studies, findings have not been universally consistent and appear dependent on disease model and metabolic status, and experimental context^34,35^. Strong SGLT1 inhibition has also been shown to abolish ischaemic preconditioning and attenuate AMPK-mediated cardioprotective signalling, whereas more selective SGLT2 inhibition preserved adaptive responses^36,37^. In addition, dual SGLT1/2 inhibition has been associated with impaired post-MI outcomes under specific experimental conditions^35^. Collectively, these observations suggest that cardioprotective effects of SGLT inhibition may be experimental context-dependent, and the absence of improved post-ischaemic recovery observed here may reflect disease-specific characteristics of severe T2D in db/db mice rather than a general lack of cardioprotective efficacy of SOTA.

The lack of improvement in post-ischaemic functional recovery may also reflect incomplete restoration of metabolic flexibility. Although SOTA improved the myocardial metabolomic profile under basal conditions, these changes do not necessarily imply restoration of substrate adaptability during stress. Short-term dual SGLT1/2 inhibition may be insufficient to restore the cellular pathways required for ischemic tolerance in the diabetic myocardium. The persistence of mitochondrial and amino acid metabolic abnormalities may limit the ability of diabetic hearts to tolerate ischemic stress^5^. Under I/R conditions, where rapid metabolic adaptation is required, persistent metabolic abnormalities may limit functional reserve and attenuate potential benefits of treatment. Together with altered stress-responsive signalling pathways, including SGLT1-associated adaptive mechanisms, this may contribute to the dissociation between improved basal metabolism and unchanged post-ischaemic recovery.

It is also possible that the cardioprotective effects of SGLT inhibition described in clinical studies are mediated in part through systemic mechanisms rather than direct myocardial metabolic changes^18,38,39^. SGLT inhibitors influence circulating substrates, hemodynamics, and renal sodium handling, factors that are absent in the isolated Langendorff heart preparation used in the present study.

Multi-organ metabolomic profiling revealed widespread metabolic disturbances across liver, kidney, and skeletal muscle in db/db mice. Although SOTA reduced the number of altered metabolites in each tissue, several abnormalities persisted despite improvement of systemic hyperglycaemia. Continued elevation of branched-chain amino acids and alterations in redox cofactors in the liver suggest persistent dysregulation of hepatic amino acid metabolism and mitochondrial redox balance^40^. Similarly, multiple metabolic abnormalities remained in the kidney. Interestingly, myocardial metabolism appeared more responsive to SOTA treatment than peripheral tissues, with more substantial restoration of energetic and glycolytic intermediates despite only partial correction of broader systemic metabolic abnormalities. These findings suggest that cardiac metabolic remodelling may exhibit differential sensitivity to SOTA treatment within the context of incomplete systemic metabolic adaptation^41^.

Taken together, the present study provides a comprehensive assessment of systemic metabolic phenotype, myocardial metabolic remodelling, cardiac function, and ischemia/reperfusion susceptibility in db/db mice. SOTA treatment improved systemic hyperglycaemia and partially reversed structural remodelling of the diabetic heart, including amelioration of diastolic dysfunction, a hallmark feature of diabetic cardiomyopathy. These changes were accompanied by selective improvements in myocardial metabolic pathways, with restoration of glycolytic intermediates and improved energetic and redox metabolites. However, broader metabolic disturbances persisted across multiple tissues, and myocardial susceptibility to ischemia/reperfusion injury remained unchanged. These findings highlight the complex relationship between systemic metabolic control, myocardial metabolic remodelling, and cardiac stress tolerance in diabetes. While SOTA improves selected metabolic pathways and cardiac structure, improvement of hyperglycaemia alone appears insufficient to fully restore metabolic and functional resilience in diabetic cardiomyopathy. Future studies are needed to determine whether longer treatment durations or strategies targeting insulin resistance and mitochondrial metabolism can provide more complete cardiometabolic protection.

Several limitations of the present study should be considered when interpreting the findings. The duration of SOTA treatment was limited to four weeks. Although this period was sufficient to reduce circulating glucose levels and induce detectable changes in myocardial metabolite profiles, longer treatment durations may be required for more pronounced systemic metabolic improvements, which may further enhance cardiac metabolism and protection against ischemia/reperfusion injury. Furthermore, the dose selected in this study (5 mg/kg/day) was intentionally conservative and within the range reported to produce biological and cardiac effects in rodent studies of SOTA and related dual SGLT1/2 inhibitors, where doses of approximately 10 mg/kg/day are commonly used in chronic cardiac models^42,43^. At 5 mg/kg/day, we observed significant reductions in hyperglycaemia accompanied by improvements in vivo cardiac function and myocardial metabolomic profiles. Future studies could explore alternative dose-duration regimens to further refine the therapeutic window. Myocardial metabolic alterations were assessed using targeted LC-MS/MS metabolomics, which quantifies steady-state metabolite concentrations but does not directly measure metabolic flux. Changes in metabolite abundance may therefore reflect alterations in production, utilisation, or compartmentalisation of metabolites, and additional studies incorporating stable isotope tracing or flux analysis would be required to more precisely define pathway activity. The ischemia/reperfusion experiments were performed using the isolated Langendorff-perfused heart model. While this preparation allows controlled assessment of intrinsic cardiac susceptibility to ischemic injury, it excludes systemic influences such as circulating hormones, substrate availability, neurohumoral signalling, and hemodynamic factors. In vivo assessed heart rate was lower in db/db mice, a recognised characteristic of this model^5^, and may influence certain echocardiographic parameters, particularly indices of diastolic function. However, heart rate did not differ between untreated and SOTA-treated db/db mice, suggesting that treatment-related improvements in cardiac function are unlikely to be driven solely by alterations in heart rate. Because SGLT inhibitors exert multiple systemic effects, including alterations in glucose handling, substrate metabolism, and renal sodium balance, the absence of these systemic factors may limit the ability of the ex vivo model to capture the full cardiometabolic effects of SOTA. Finally, the db/db mouse model represents a severe form of obesity-driven T2D resulting from leptin receptor deficiency. Although this model recapitulates many features of human metabolic disease, including insulin resistance and hyperglycaemia, the genetic basis and severity of the phenotype may differ from typical human diabetes. Therefore, caution should be exercised when extrapolating these findings directly to clinical populations.

## Methods

### Animals

Commercially available type 2 diabetic mice (db/db mouse, The Jackson Laboratory homozygous BKS.Cg-Dock7^m^ +/+ Lepr^db^/J, male, Charles River, Italy) were purchased at 12 weeks of age (*n* = 27). Age matched lean C57BL/6 J mice (Charles River, UK) were purchased as controls (*n* = 13). All animals were aged until 20 weeks. Animals were kept under pathogen-free conditions, 12 h light–dark cycle, controlled temperature (20–22 °C), with access to standard chow diet (irradiated PicoLab® Mouse Diet 20 EXT, 5R58) and water *ad libitum*. Circulating glucose (tail sample, Accu-Check, Roche) and body weight were monitored weekly.

### Ethics statement

This investigation was approved by the Animal Welfare and Ethical Review Board at Queen Mary University of London and conforms to the UK Animals (Scientific Procedures) Act 1986 (Home Office Project Licence PP9914604) incorporating Directive 2010/63/EU of the European Parliament and conforms to European Convention for the Protection of Vertebrate Animals Used for Experimental and Other Scientific Purposes (Council of Europe, No 123, Strasbourg, 1985).

### Drug treatment experimental protocol

db/db mice (*n* = 14) were randomised to receive SOTA (MedChem Express, UK) at 5 mg/kg/day given in drinking water (Fig. 1, schematic summary of the experimental protocol). Drug treatment was initiated at 16 weeks age and given for the remaining 4 weeks of study. Water consumption was monitored before and after initiation of SOTA treatment to ensure water consumption was unchanged by addition of drug (pre-drug 13 ± 0.7 ml/day, post-drug 14 ± 0.5 ml/day, *p* > 0.05). At the end of the protocol, mice were euthanised by intraperitoneal phenobarbital injection and tissues [hearts, liver, kidney and skeletal muscle (gastrocnemius and soleus)] were rapidly excised and immediately freeze-clamped using Wollenberger tongs for metabolic profiling.

### Echocardiography

M-mode and Doppler echocardiography was performed in 20-week lean controls, db/db and SOTA treated db/db mice at the end of the drug treatment protocol as previously described^2,5^. Echocardiography images were recorded using a Vevo-3100 imaging system with a 40 MHz linear probe (VisualSonics, Toronto, Canada). Morphological measurements were taken in the parasternal short axis view at the level of the papillary muscles and ejection fraction was calculated from M-mode images. For assessment of diastolic function, pulsed-wave Doppler was performed in the apical four-chamber view to obtain transmitral LV inflow velocities, including early (E) and late (atrial, A) peak filling velocities. Additionally, tissue Doppler imaging in the apical four-chamber view was used to obtain mitral annular velocities: early diastolic (E′) and late diastolic/atrial contraction (A′). Analysis was performed using VevoLab 5.5.1 software. Cardiac function was assessed blinded to the phenotype.

### Metabolomics

Cardiac metabolism was assessed using targeted LC-MS/MS to enable high-sensitivity quantification of key intermediates involved in central carbon metabolism. In contrast, peripheral tissue metabolism was analysed using ¹H NMR spectroscopy to provide broader, unbiased metabolite profiling suitable for comparative multi-tissue analysis.

For LC-MS/MS analysis, frozen pulverised cardiac tissue samples ( ~ 15 mg) were extracted using a buffer (50% Methanol, 30% Acetonitrile, 20% ultrapure water, and 50 ng/ml 1 mM HEPES solution) as previously described in refs. ^1,5,44^. LC-MS analysis was performed by the metabolic flux analysis facility of the Barts Faculty of Medicine and Dentistry using a Q Exactive Quadrupole-Orbitrap mass spectrometer coupled to a Vanquish UHPLC system (Thermo Fisher Scientific).

Frozen, pulverised skeletal muscle (gastrocnemius and soleus), liver and kidney samples ( ~ 45 mg) were subject to methanol/water/chloroform phase extraction and analysed using ^1^H NMR high-resolution spectroscopy as previously described in refs. ^1,2^.

### Plasma analysis

Blood samples were collected at the time of experimental endpoint into heparinised tubes. Plasma biochemical profiling was carried out by the MRC Mouse Biochemistry Laboratory (Addenbrookes NHS Hospital, Cambridge).

### Langendorff heart perfusions

Mice were euthanised by intraperitoneal phenobarbital injection, hearts rapidly excised, cannulated and perfused as a standard Langendorff preparation as previously described in refs. ^1,5^. Krebs-Henseleit (KH) perfusion buffer was continuously gassed with 95% O_2_/5% CO_2_ (pH 7.4, 37 °C) containing (in mM): NaCl (116), KCl (4.7), MgSO_4_.7H_2_O (1.2), KH_2_PO_4_ (1.2), NaHCO_3_ (25), CaCl2 (1.4), and glucose (11). Cardiac function was assessed using a fluid-filled cling-film balloon inserted into the LV connected via a line to a pressure transducer and a Powerlab system (AD Instruments). Functional parameters were recorded using LabChart software v.7 (AD Instruments) throughout the experiment. LV developed pressure (LVDP) was calculated from the difference between systolic and diastolic pressure.

### Ischemia/reperfusion protocol

Langendorff perfused hearts were subjected to global ischemia reperfusion as previously described in refs. ^1,5^. In brief, after 20 min of equilibration, Langendorff crystalloid KH buffer perfused hearts were subject to 20 min of global normothermic ischemia (37 °C) and 2 h of reperfusion (37 °C). At the end of the protocol myocardial infarct size was quantified using triphenyltetrazolium chloride (TTC) staining. Hearts were perfused for 1 min with 1% TTC in KH Buffer followed by 10 min incubation in 1% TTC-KH. Hearts were sectioned (mouse heart gauge, Zivic instruments, USA) and infarct field was quantified using Fiji-ImageJ Software.

### Principal component analysis

Principal component analysis (PCA) was performed as an unsupervised multivariate method to reduce dimensionality and assess clustering patterns within the ^1^H NMR metabolomics data. Missing values were low across features ( < 50%) and were imputed using limit of detection approach (1/5 of the minimum positive value). Data were subsequently normalised to AUC, log transformed and auto scaled. Metabolites contributing to group separation were identified using PCA loading vectors. PCA analysis was conducted using R studio (v.4.4.2).

### Statistical analysis

Data are presented as mean ± SEM. Normality of data distribution was examined using Shapiro-Wilk’s normality test. One-way ANOVA with Holm-Šídák post-hoc analysis was used for multiple comparisons, or Kruskal-Wallis test with Dunn’s post-hoc analysis when data was non-normally distributed. Two-way repeated measures ANOVA was used where applicable, with post-hoc analysis using Fisher’s LSD test displayed when interaction was significant. Differences between groups were considered significant at *p* < 0.05. Statistical analysis was conducted using GraphPad Prism software (v.10.40.1).

## Supplementary information

Supplementary Information

Supplementary Data

## Data Availability

All source data are provided in the accompanying supplementary materials.
