# Supplementary material for: Dual SGLT1/2 inhibition with sotagliflozin improves cardiac function and metabolism in type 2 diabetic mice
Source: NPJ Metab Health Dis. 2026 Aug 5;4:33. doi: 10.1038/s44324-026-00119-z (PMC13443782; doi:10.1038/s44324-026-00119-z)
Supplement: Supplementary file 1 [file 44324_2026_119_MOESM1_ESM.docx]

**Dual SGLT1/2 inhibition with sotagliflozin improves cardiac remodeling and myocardial metabolism in db/db diabetic mice**

**Supplementary Materials**

**Fig. S1:** Cardiac morphology assessed by echocardiography. **(A-B)** Interventricular septum wall thickness in diastole (A) and systole (B). **(C-D)** Left ventricular posterior wall (LVPW) thickness in diastole (C) and systole (D). Analysed by one-way ANOVA with Holm-Šídák post-hoc analysis displayed (A, B, D) or Kruskal-Wallis test with Dunn’s post-hoc analysis displayed (C), *p<0.05. Lean n=6, db/db n=9, db/db + SOTA n=9.

**Fig. S2:** Recovery of dP/dt max during reperfusion following 20 minutes global ischemia. Analysed by two-way repeated measures ANOVA, significant interaction of time x group (p=0.05), post-hoc comparisons using Fisher’s LSD test displayed for each time point (*p<0.05 lean vs. db/db; #p<0.05 lean vs. db/db + SOTA). Lean (n=4), db/db (n=7), db/db + SOTA (n=7).
